# The impact of facemask on patients with COPD: A systematic review and meta-analysis

**DOI:** 10.3389/fpubh.2022.1027521

**Published:** 2022-11-16

**Authors:** Xuwen Chen, Changqing Zhang, Sani Ibrahim, Shunyu Tao, Xiaoli Xia, Yi Li, Caiyun Li, Feiyan Yue, Xinhua Wang, Shisan Bao, Jingchun Fan

**Affiliations:** ^1^School of Public Health, Center for Laboratory and Simulation Training, Centre for Evidence-Based Medicine, Gansu University of Chinese Medicine, Lanzhou, China; ^2^Department of Radiology, Gansu Provincial People's Hospital, Lanzhou, China; ^3^School of Public Health, The University of Sydney, Camperdown, NSW, Australia; ^4^Department of Geriatrics, Affiliated Hospital of Gansu University of Chinese Medicine, Lanzhou, China; ^5^Department of Respiratory Cadres, Gansu Provincial People's Hospital, Lanzhou, China

**Keywords:** chronic obstructive pulmonary disease (COPD), facemasks, systematic review, meta-analysis, COVID-19

## Abstract

**Background:**

Since the emergence of COVID-19, mandatory facemask wearing has been implemented around the world to prevent viral transmission, however, the impact of wearing facemasks on patients with COPD was unclear.

**Methods:**

The current study undertakes a systematic review and meta-analysis of a comprehensive literature retrieval from six databases, based on the pre-determined eligibility criteria, irrespective of language. The risk of bias was assessed using an established instrument. We primarily focused on analyzing ETCO_2_, SpO_2_, and heart and respiratory rates, and also considered the impacts on physiological and exercise performance. A descriptive summary of the data and possible meta-analysis was performed. Forest plots were generated to pool estimates based on each of the study outcomes.

**Results:**

Of the 3,751 publications considered, six publications were selected for a systematic review and two publications were included for meta-analysis, however, the quality of these six studies was relatively low overall. In the case of inactivity, the facemask wearing COPD cohort had higher respiratory rates than that of the non-facemask wearing cohort (MD = 1.00 and 95% CI 0.47–1.53, *P* < 0.05). There was no significant difference in ETCO_2_ (MD = 0.10 and 95% CI −1.57–1.78, *P* > 0.05) and heart rate (MD = 0.40 and 95% CI −3.59–4.39, *P* > 0.05) nor SpO_2_ (MD = −0.40 and 95% CI −0.84–0.04, *P* > 0.05) between the COPD patients with and without facemasks. Furthermore, it was observed that the only significant differences between the COPD patients with and without facemasks undertaking different activities were FEV1 (%) (MD = 3.84 and 95% CI 0.14–7.54, *P* < 0.05), FEV1/FVC (%) (MD = 3.25 and 95% CI 0.71–5.79, *P* < 0.05), and blood lactate (MD = −0.90 and 95% CI −1.73 to −0.07, *P* < 0.05).

**Conclusion:**

Wearing facemasks decreased the exercise performance of patients with COPD, however, it had minimal impact on physiological indexes. Further investigations will be performed on the high-quality data from randomized control studies.

**Systematic review registration:**

https://www.crd.york.ac.uk/PROSPERO/display_record.php?RecordID=326265, identifier: CRD42022326265.

## Introduction

Chronic obstructive pulmonary disease (COPD) is a chronic inflammation in the respiratory system that causes obstructed airflow from the lungs (1). The global prevalence was 10.3% (aged between 30 and 79 years) in 2019 with mortality of ~3.2 million (2), which accounted for 81.7% of all deaths from chronic respiratory diseases (3). COPD is the third most common cause of mortality worldwide and a leading cause of chronic morbidity and hospitalization with a significant economic burden (4). The high mortality and morbidity of COPD are likely due to a combination of the increased number of smokers, aging population, lack of awareness of the long-term health consequences, and inadequate access to early diagnosis in society (5, 6). More recently, patients with COPD have been advised to wear facemasks in public to prevent and/or minimize the spread of COVID-19 during the pandemic and to minimize the consequential comorbidity of COPD and COVID-19 (7).

Facemasks are used for preventing/minimizing airborne pathogen transmission or pollution by the general public and healthcare personnel (8), however, which are not routinely used as personal protective equipment. A facemask is a loose-fitting, disposable device that creates a physical barrier between the upper respiratory tract and potential contaminants in the air. There are different types of facemasks, including surgical facemasks, barrier face coverings, N95 respirators, and other filtering facepiece respirators.

It is well-known that inhaled air quality is a causal factor in exacerbating COPD (9, 10). This is consistent with the finding that the rate of hospitalization of COPD patients with acute exacerbation decreased significantly during the period of the COVID-19 pandemic in China. COPD patients usually present with cough, worsening dyspnoea, progressive exercise intolerance, sputum production, and alteration in mental status (11). In addition, acute exacerbation of COPD presents with the aggravation of dyspnoea, increased sputum volume, or purulent sputum, which is often accompanied by fever, cough aggravation, or wheezing (11). This is likely due to the imposition of mandatory facemask wearing in all public settings during the pandemic to minimize potential pathogenic transmission (12–14) and inadvertently improved the quality of inhaled air. The advice from medical practitioners for COPD patients to wear facemasks was intended to minimize potential air pollution and the consequential exacerbation of the chronic inflammation in the respiratory system, rather than for any medical intervention (15). However, the potential impact of wearing facemasks on COPD patients was uncertain as facemasks increase dead space in the respiratory system with potential deteriorating outcomes (16). Therefore, we aimed to determine the impact of wearing facemasks on physiological indexes and activities on patients with COPD by undertaking a systematic review and meta-analysis to provide insights for clinical guidance as well as public health concerns on wearing facemasks while carrying on different activities, particularly among patients with COPD.

## Methods

### Search strategy and selection criteria

We prospectively registered this systematic review and meta-analysis on PROSPERO (ID: CRD42022326265) and followed the guidelines of Preferred Reporting Items for Systematic Reviews and Meta-Analysis (PRISMA). We searched six electronic databases from their inception up to 12 May 2022, regardless of language or publication date, using a comprehensive strategy to select eligible studies, including the Cochrane Library, Embase, PubMed, Web of Science, the Chinese Biomedical Database (Sino-Med), and China National Knowledge Infrastructure (CNKI). An example search strategy for the PubMed database is presented (Table 1). All relevant references were retrieved for further verification. Two reviewers independently screened all abstracts and titles for relevance. The full text of articles that met the selection criteria was collected for assessment.

**Table 1:** Search strategy (PubMed).

#1 Search	“Pulmonary Disease, Chronic Obstructive” [Mesh]
#2 Search	(((((((Chronic Obstructive Lung Disease [Title/Abstract]) OR (Chronic Obstructive Pulmonary Diseases[Title/Abstract])) OR (COAD[Title/Abstract])) OR (COPD[Title/Abstract])) OR (Chronic Obstructive Airway Disease[Title/Abstract])) OR (Chronic Obstructive Pulmonary Disease[Title/Abstract])) OR (Chronic Airflow Obstructions[Title/Abstract])) OR (Chronic Airflow Obstruction[Title/Abstract])
#3 Search	#1 OR #2
#4 Search	“Facemasks” [Mesh] OR “N95 Respirators” [Mesh]
#4 Search	(((((facemask[Title/Abstract]) OR (facemasks[Title/Abstract])) OR (facemask [Title/Abstract])) OR (facemasks[Title/Abstract])) OR (face-facemask [Title/Abstract])) OR(face-facemasks[Title/Abstract]) OR (((((((N95 Respirator [Title/Abstract]) OR (N95 Face Facemasks[Title/Abstract])) OR (N95 Face Facemask [Title/Abstract])) OR (N95 Facemasks[Title/Abstract])) OR (N95 Facemask [Title/Abstract])) OR (N95 Filtering Facepiece Respirators [Title/Abstract])) OR (N95 FFRs[Title/Abstract])) OR (N95 FFR[Title/Abstract])
#6 Search	#4 OR #5
#7 Search	#3 AND #6

### Inclusion and exclusion criteria

The selection criteria included the following: (1) All studies related to patients with COPD and masks; (2) Patients with COPD diagnosed according to the definition by the Global Initiative for Chronic Obstructive Lung Disease (17) and age, sex, and disease severity were disregarded; (3) Wearing facemasks, defined by World Health Organization (18), as a means of prevention, regardless of the type of masks; (4) No-facemasks were used as a control. The types of masks were disregarded from our current selected published studies; (5) Types of studies including randomized trials (including cluster-randomized trials) and non-randomized trials; and (6) Outcome indicators were related to the physiology and activity of patients with COPD, including ends tidal carbon dioxide, respiratory rate, heart rate, oxygen saturation, pulmonary function, blood pressure, blood lactate, oxygen partial pressure, carbon dioxide partial pressure, minute ventilation, inspiratory time, 6-min walking test (6 MWT), expected relative exercise capacity, and work rate. Data for different outcome indicators can be extracted.

The exclusion criteria included the following: (1) animal studies; (2) the articles were meta-analysis, review, and/or conference abstracts; (3) the studies had incomplete data; (4) the study had no access to full text; (5) facemasks used as interfaces for non-invasive positive-pressure ventilation, including oronasal masks and nasal masks; and (6) duplication.

### Data extraction and quality assessment

Two researchers extracted data independently using pre-designed forms, including the demographics, methods, and results in measurements. Discrepancies were resolved by consulting the third senior researcher to arrive at a consensus. Authors of studies with incomplete data were contacted for full text, however, the papers were excluded if no relevant data was ultimately obtained. The Cochrane Risk of Bias 2 Tool was used to assess randomized controlled trials and crossover trials (19). The suggested risk of bias criteria proposed by the Effective Practice and Organization of Care reviews group of the Cochrane collaboration was used to evaluate non-randomized controlled trials (20). For each included literature involving trials, “low risk,” “unclear risk,” and “high risk” were judged for each item.

The quality of evidence for the outcomes of the meta-analysis has been assessed using the Grading of Recommendations Assessment, Development and Evaluation (GRADE) approach. Using the GRADEpro GDT online tool, the evidence was categorized into five aspects: study limitations, inconsistency of results, indirectness of evidence, imprecision, and publication bias, using “very low,” “low,” “moderate,” or “high” judgments for each evidence level (21).

### Statistics analysis

Statistical analysis was performed, using Review Manager 5.4 software provided by the Cochrane Collaboration. Mean Difference (MD) was used as the effect indicator. The 95% confidence interval (CI) was calculated. The baseline value and final value after the intervention was used as the main effect parameters, where an assumed correlation coefficient (Corr) was set to 0.5. The *Q* statistic and *I*^2^ index were used to evaluate the heterogeneity between studies. The combined effect size was calculated, using the fixed-effect model. It was considered a significant difference in the heterogeneity test results when *P* < 0.1 and *I*^2^ ≥ 50%. The combined effect size was calculated, using the random effect model.

## Results

### Search results

**Figure 1:**
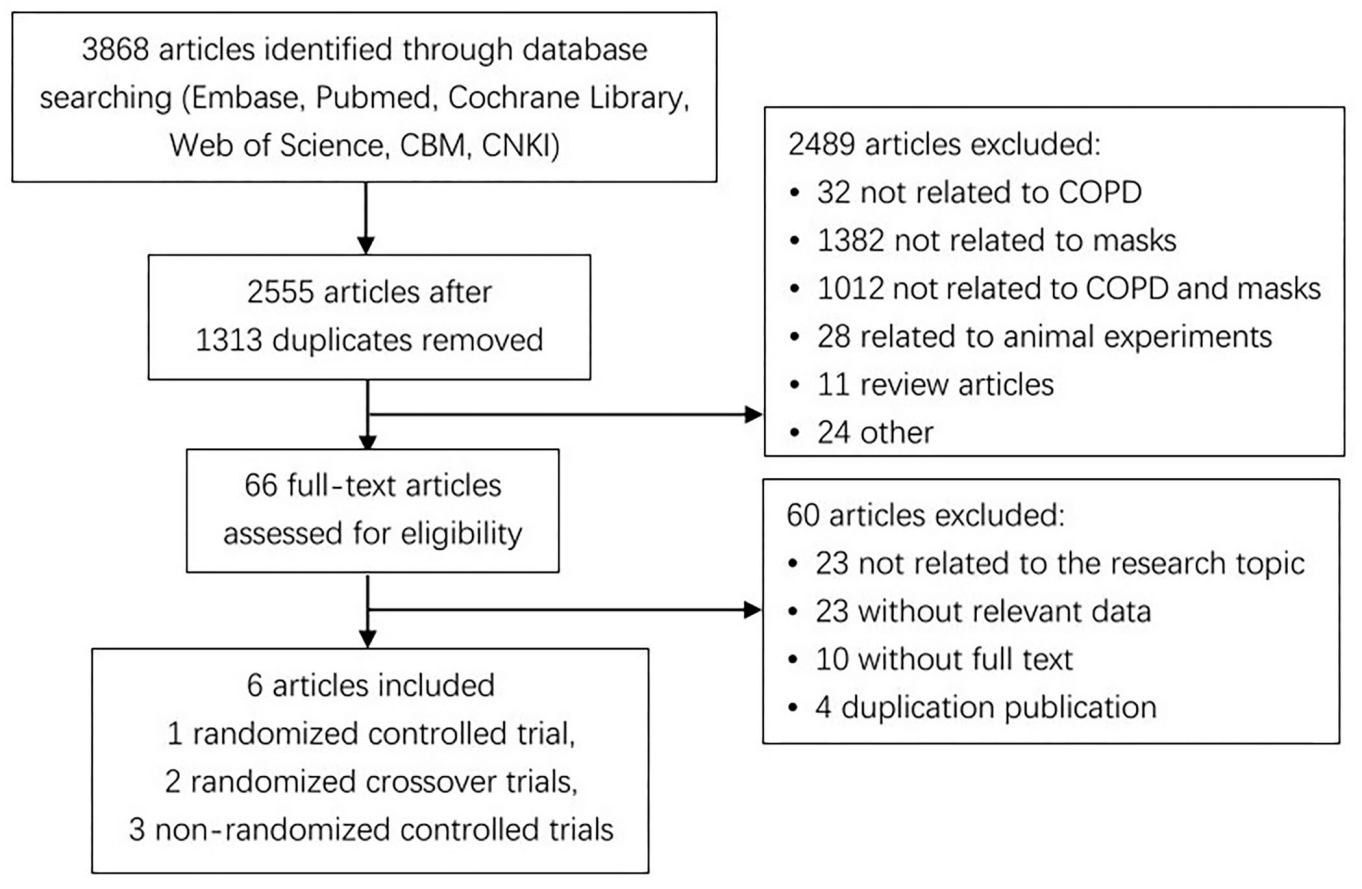
STROBE diagram of the study's selection process.

The initial screening of titles and abstracts only yielded 3,868 records. Sixty-six validated papers were identified after removing 1,313 duplicates and excluding 2,489. Finally, only six publications published between 2010 and 2021 met the strict inclusion criteria. Three out of the six studies were randomized controlled trials (22–24), including two crossover designs (23, 24); whereas the remaining three studies were non-randomized controlled trials (15, 25, 26). A flow diagram of the literature search and related screening process is illustrated below (Figure 1).

### Description of the included studies

There were 313 people diagnosed with COPD from 6 included studies that wore facemasks, including 3M facemasks (22), common facemasks in cardiopulmonary exercise tests (23), N95 facemasks (15, 24), dual cartridge half-face facemasks (24), disposable non-filter medical facemasks (25), and surgical facemasks (26). The patients from the three studies (15, 25, 26) carried out a 6-min walk test (6 MWT). One study was related to the normal physical activity of patients with COPD (22), and another study required the patients to perform a maximum exercise test on a cycle ergometer (23). Only the patients with COPD were required to perform different exercises involving eight movements at the level of sedentary, mild exertion, and moderate exertion (24). The key characteristics of these six publications are summarized in Table 2.

**Table 2:** Characteristics of included studies.

**References**	**Country**	**Publication year**	**Study design**	**Males, No. (%)**	**Sample size**	**Age (x ±*s*)**	**Exercise protocol**	**Facemasks**	**Outcomes (effect size)**
Harber et al. (24)	Austria	2010	Randomized crossover trial	12 (86)	14	53.8 ± 4.7	A series of simulated-work tasks	HFM N95	HFM vs. N95:Ve: −0.33 [−5.08,4.42] Ti: 0.03 [−0.02,0.08]
Dong et al. (22)	China	2016	RCT	82 (59)	140	65.3 ± 2.5	Outgoing normal activities	3M 9010 protective facemask	Facemask vs. no-facemask: FEV_1_(%): 3.84 [0.14, 7.54] FEV_1_/FVC(%): 3.25 [0.71, 5.79]
Neunhäuserer et al. (23)	USA	2017	Randomized crossover trial	18 (67)	27	63.3 ± 6.5	Maximum exercise test on a cycle ergometer	A common facemask used in cardiopulmonary exercise tests	Facemask vs. no-facemask (at exhaustion): HR_max_: −1.90 [−10.84, 7.04] SBP_max_: −6.80 [−24.37, 10.77] Lactate_max_: −0.90 [−1.73, −0.07] EREC: −6.80 [−15.63, 2.03] P_max_: −9.90 [−28.20, 8.40] Facemask vs. no-facemask (at the Borg rating of perceived exertion 12–14/20): HR: 4.00 [−3.55, 11.55] Lactate: −0.20 [−0.58, 0.18] P: −4.20 [−16.53, 8.13]
Kyung et al. (15)	Korea	2020	Non-RCT	91 (94)	97	68.0 ± 6.5	6 MWT 10-min rest	N95	Facemask + 6MWT vs. no-facemask + 6MWT: ETCO_2_: 1.50 [−0.61, 3.61] RR: 2.40 [0.76, 4.04] HR: 4.70 [−0.30, 9.70] SpO_2_: −0.80 [−1.56, −0.04] SBP: 0.40 [−4.36, 5.16] DBP: −0.80 [−4.13, 2.53] Facemask + 10-min rest vs. no-facemask: ETCO_2_: 0.9 [−1.13, 2.93] RR: 1.00 [0.47, 1.53] HR: 0.40 [−3.59, 4.39] SpO_2_: −0.40 [−0.84, −0.04] SBP: 1.90 [−2.39, 6.19] DBP: 2.70 [−0.34, 5.74]
Just et al. (25)	Germany	2021	Non-RCT	–	20	–	6 MWT	Disposable, non-filtering medical facemasks	Facemask + 6MWT vs. no-facemask + 6MWT: 6MWD: −7.40 [−72.52, 57.72] SaO2: 0.40 [−3.70,4.50]
Samannan et al. (26)	USA	2021	non-RCT	15 (100)	15	71.6 ± 8.7	6 MWT 30-min rest	A surgical facemask	Facemask + 30-min rest vs. no-facemask: ETCO2: −1.63 [−4.62, 1.36] RR: 1.03 [−2.53, 4.59]

### Assessment of quality and risk of bias

All studies were identified as having “some concerns” when the quality of a randomized controlled trial and two randomized crossover trials were assessed by the Cochrane Risk of Bias 2 Tool (19). The bias for all studies (22–24) was due to the randomization process and deviations from the intended intervention. Based on the suggested risk of bias criteria for EPOC reviews (20), all non-randomized controlled studies (15, 25, 26) were scored “high risk” in the category of “random sequence generation” (Table 3). There was no serious indirectness that existed. However, due to the limitations of non-randomized controlled trials and the small sample size, the overall quality of evidence was still graded as being low (Table 4).

**Table 3:** Quality assessment of included studies.

**RCTs**	**1**	**2**	**3**	**4**	**5**	**The overall risk of bias**
Dong et al. (22)	**	**	*	*	**	**
Harber et al. (24)	**	**	*	*	**	**
Neunhäuserer et al. (23)	**	**	*	*	*	**
**Non-RCTs**	**1**	**2**	**3**	**4**	**5**	**6**	**7**	**8**	**9**
Just et al. (25)	***	**	**	*	*	*	**	*	**
Kyung et al. (15)	***	**	*	*	**	*	**	*	**
Samannan et al. (26)	***	**	*	*	**	*	*	*	**

1. Randomization process; 2. Deviation from intended intervention; 3. Missing outcome data; 4. Measurement of outcome; 5. Selection of reported results. ^*^, Low risk; ^**^, Some concerns; ^***^, High risk.

1. Random sequence generation; 2. Allocation concealment; 3. Baseline outcome measurements similar; 4. Baseline characteristics similar; 5. Incomplete outcome data; 6. Knowledge of the allocated interventions adequately prevented during the study; 7. Protection against contamination; 8. Selective outcome reporting; 9. Other risks of bias.

^*^, Low risk; ^**^, Unclear; ^***^, High risk.

**Table 4:** Quality assessment using the GRADE approach.

**Certainty assessment**							**No of patients**		**Effect**		**Certainty**	**Importance**
**No of studies**	**Study design**	**Risk** **of bias**	**Inconsistency**	**Indirectness**	**Imprecision**	**Other considerations**	**Facemask**	**No-facemask**	**Relative (95% CI)**	**Absolute (95% CI)**		
**What is the effect of facemasks on ETCO**_**2**_ **in COPD patients? (assessed with: ETCO2)**												
2	Non-randomized controlled trial studies	Serious^a^	Not serious	Not serious	Serious^b^	None	105	112	–	MD 0.10 mm Hg higher (1.57 lower to 1.78 higher)	⊕○○○ Very low	Not important
**What is the effect of facemasks on RR in COPD patients? (assessed with: RR)**												
2	Non-randomized controlled trial studies	Serious^a^	Not serious	Not serious	Serious^b^	None	105	112	–	MD 1.00 breaths/min higher (0.47 higher to 1.53 higher)	⊕○○○ Very low	Not important

Author(s): XWC, CQZ, and JCF.

Question: What is the effect of facemasks on physiological indexes in COPD patients?

Setting: COPD patients.

CI, confidence interval; MD, mean difference.

a Downgraded by 1 for risk of bias: pooled result includes 2 non-RCT (high risk of selection bias).

b Downgraded by 1 for imprecision: 2 trials with small sample size (n = 97; n = 15).

### Effects of interventions

#### The primary outcomes

##### Ends tidal carbon dioxide (ETCO_2_)

**Figure 2:**
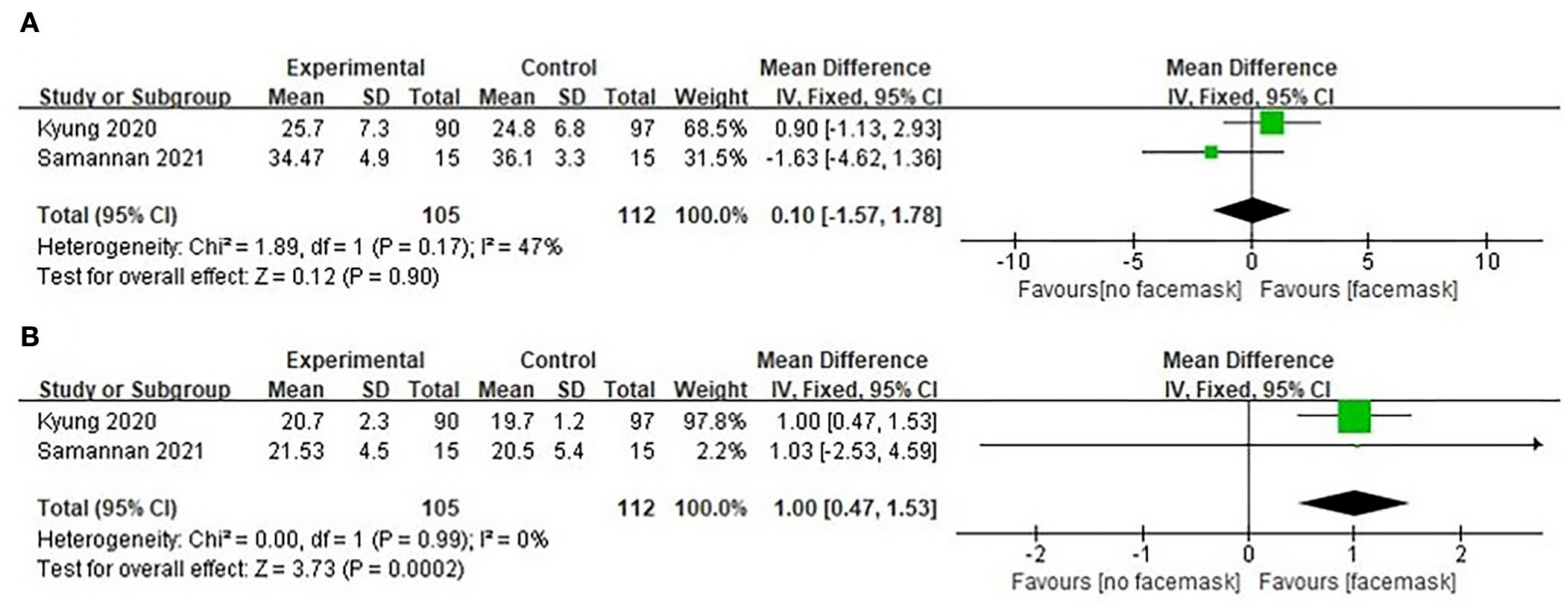
Forest plot of facemask intervention studies (facemask vs. no-facemask) **(A)** ETCO2 and **(B)** RR.

The data of two publications (15, 26) were used for a meta-analysis of ETCO_2_, illustrating that there was no significant difference in ETCO_2_ between COPD patients with and without facemasks at rest (*n* = 112) (MD = 0.10 and 95% CI −1.57–1.78, *P* > 0.05; Figure 2A). The quality of evidence assessed was very low, according to the GRADE criteria and the reasons for the downgrade included the study limitations and imprecision (Table 4). In addition, there was no significant difference in ETCO_2_ between the COPD patients (*n* = 97) with and without facemasks after 6 MWT (MD = 1.50 and 95% CI −0.61–3.61, *P* > 0.05) (15).

##### Respiratory rate

Two studies (15, 26) pooled results for meta-analysis of respiratory rate (*n* = 112). The respiratory rate increased significantly at rest from COPD patients with facemasks compared to COPD patients without facemasks (MD = 1.00 and 95% CI 0.47–1.53, *P* < 0.05; Figure 2B). Due to selective bias and a small sample size, the quality of evidence assessed was still very low, according to the GRADE criteria (Table 4). Following 6 MWT, the respiratory rate was significantly higher in COPD patients with facemasks compared to those without (MD = 2.40 and 95% CI 0.76–4.04, *P* < 0.05) (*n* = 97) (15).

##### Heart rate

There was no significant difference in heart rate from COPD patients (*n* = 97) with facemasks following 6 MWT and then 10 min rest, compared to that from the same cohort at the baseline (MD = 0.40 and 95% CI −3.59–4.39, *P* > 0.05) (15). There was no significant difference in heart rate between the COPD patients with facemasks and without facemasks (MD = 4.70 and 95% CI −0.30–9.70, *P* > 0.05) (*n* = 97) following 6 MWT (15). Moreover, there was no significant difference in the maximum heart rate between COPD patients with and without facemasks (MD = −1.90 and 95% CI −10.84–7.04, *P* > 0.05) (23). There was no significant difference in the Borg Scale Rate of Perceived Exertion at 12–14 (Borg-RPE) between COPD patients with and without facemasks (MD = 4.00 and 95% CI −3.55–11.55, *P* > 0.05) (23).

##### Oxygen saturation (SpO_2_ and SaO_2_)

SpO_2_ was significantly higher in COPD patients (*n* = 97) without facemasks than those with facemasks at rest (MD = −0.80 and 95% CI −1.56 to −0.04, *P* < 0.05) (15). However, there was no significant difference in SaO_2_ between the COPD patients (*n* = 20) with and without facemasks (MD = 0.40 and 95% CI −3.70–4.50, *P* > 0.05) after a 6 MWT (25).

#### Secondary outcomes

##### Pulmonary function

Pulmonary function, including FEV_1_ and FEV_1_/FVC, was evaluated in patients with COPD (22). These patients with COPD were advised to wear facial masks to minimize haze inhalation due to air pollution. Significantly lower FEV1 and FEV1/FVC were observed in COPD patients with an acute exacerbation who did not wear facemasks than those COPD patients with facemasks [FEV1 (%) MD = 3.84 and 95% CI 0.14–7.54, *P* < 0.05; FEV1/FVC (%) MD = 3.25 and 95% CI 0.71–5.79, *P* < 0.05]. However, no information was available about the length of time the patients did not wear facemasks.

##### Blood pressure

There was no significant difference in the systolic blood pressure of COPD patients with and without facemasks following the maximum exercise test (MD = −6.80 and 95%CI −24.37–10.77, *P* > 0.05) (23). This finding is consistent with other studies (15) illustrating that there was no significant variance in systolic blood pressure between COPD patients with and without facemasks following 6 MWT and 10-min rest compared to the baseline (MD = 1.90 and 95% CI −2.39–6.19, *P* > 0.05). No significant difference in systolic blood pressure was observed between COPD patients with and without facemasks following 6 MWT (MD = 0.40 and 95% CI −4.36–5.16, *P* > 0.05). In addition, there was no significant difference in diastolic blood pressure between COPD patients with and without facemasks at rest (MD = 2.70 and 95% CI −0.34–5.74, *P* > 0.05) nor after 6 MWT (MD = −0.80 and 95% CI −4.13–2.53, *P* > 0.05) (15).

##### Blood lactate

Blood lactate levels from COPD patients without facemasks were significantly higher than these COPD patients with facemasks at exhaustion (MD = −0.90 and 95% CI −1.73 to −0.07, *P* < 0.05) (23). However, there was no significant difference in blood lactate levels between COPD patients with and without facemasks at the intensity of Borg-RPE 12–14 (MD = −0.20 and 95% CI −0.58–0.18, *P* > 0.05) (23).

##### Minute ventilation and inspiratory time

Changes were reported in minute ventilation (Ve) and inspiratory time (Ti) from patients with COPD when wearing dual cartridge half-face facemasks and N95 facemasks for different exercises (24). However, there was no significant difference in minute ventilation nor inspiratory time from COPD patients with and without facemask (Ve MD = −0.33 and 95% CI −5.08–4.42, *P* > 0.05; Ti MD = 0.03 and 95% CI −0.02–0.08, *P* > 0.05).

##### Six-Minute walking test (6 MWT)

There was no significant difference of 6 MWT between the COPD patients with facemasks and those without facemasks (MD = −7.40 and 95% CI −72.52–57.72, *P* > 0.05) (25).

##### Expected relative exercise capacity

There was no significant difference in EREC of patients with COPD at exhaustion with and without facemasks (MD = −6.80 and 95% CI −15.63–2.03, *P* > 0.05) (23).

##### Work rate

There was no significant difference in the maximum working rate between COPD patients with and without facemasks at exhaustion (MD = −9.90 and 95% CI −28.20 to 8.40, *P* > 0.05) (23). At Borg-RPE 12–14, the difference in working rate was also not significant (MD = −4.20 and 95%CI −16.53–8.13, *P* = 0.50) (23).

## Discussion

In the current study, we determined the impact of facemask wearing on patients with COPD based on six randomized and non-randomized controlled studies. Our meta-analysis demonstrated that a higher respiratory rate was detected in COPD patients with facemasks than in those without facemasks. FEV1 and FEV1/FVC (%) were also higher, however, blood lactate was lower at exhaustion in COPD patients with facemasks compared to those without.

In addition, our meta-analysis on two non-randomized controlled trials showed that respiratory rates, but not ETCO_2_, of COPD patients with facemasks increased at rest. Increased respiratory rates in the COPD cohort at rest may be caused by the discomfort and unfamiliarity of wearing facemasks. The facemasks may cause physical reactions, for example, increased afferent impulses from the highly thermosensitive area on the face covered by the facemask or from the increased temperature of the inhaled air. In addition, the use of facemasks may lead to psychological responses, such as anxiety, claustrophobia, or affective responses to the perceived difficulty in breathing (27). However, the increased respiratory rate from COPD patients with facemasks maybe also due to increased dead space at rest and during exercise (15).

In addition, only increased respiratory rate was observed in the COPD cohort with facemasks at rest, but no material variance was noted in ETCO_2_, heart rate, and SpO_2_. This may be due to the limited experimental time of testing the impact of facemasks. The longest duration of wearing facemasks for the test was only 30 min, which is unlikely to be sufficient to impact parameters other than respiratory rate. Thus, such data may not reflect real life, and further studies will need to be undertaken to determine the longer-term impacts. Our speculation is supported by other studies that have shown that there was no adverse physiological effect after wearing an N95 mask continuously for 1 h. However, headaches and peak transcutaneous CO_2_ levels > 50 mm Hg were associated with the continuous use of N95 masks for over 4 h (28). Furthermore, there was a significant difference in chest tightness and breath resistance between healthy young individuals with and without facemasks (29). This is in line with the finding from Shui et al. (30) demonstrating that wearing facemasks at rest prolonged inspiratory time but reduced minute ventilation and respiratory rates compared with the non-facemask wearing group.

Most of the studies from healthy people measured the indicators of physiological and exercise performance following 6 MWT and other active conditions (31), showing that facemasks had minimal impact on physiological variables. Radtke et al. (32) reported that heart and respiratory rates were increased in people aged between 50 and 83 years with facemasks following 6 MWT, compared to those without facemasks. However, there was no significant difference in respiratory and heart rates, comparing different types of facemasks. In addition, wearing different facemasks had no effect on oxygen saturation in healthy adults following 6 MWT (33–35). Our explanation is that there is a compensatory mechanism in the healthy cohorts with facemasks by increasing respiratory and heart rates to saturate oxygen (33, 36).

Further comparison of the respiratory rate and oxygen saturation of COPD cohorts with and without facemasks following 6 MWT illustrated that there was only a trend of increased respiratory rate and decreased oxygen saturation. Increased ETCO_2_ was observed in the healthy cohort with facemasks during exercise (37), which may be attributed to rebreathing of exhaled air (38). However, increased ETCO_2_ from COPD patients with facemasks may be more pronounced, causing more obvious hypercapnia, particularly during intense exercise (39).

Surprisingly, Dong et al. (22) demonstrated that FEV_1_% and FEV_1_/FVC were significantly increased in COPD patients with facemasks, compared to those without. The purpose of wearing facial masks for this particular cohort was to protect from environmental pollution. The increased FEV_1_% and FEV_1_/FVC may be partially due to the anti-haze effect of facemasks and improved air quality at the time. However, the pulmonary function in the healthy cohort with facemasks was significantly reduced compared to those without facemasks regardless of environmental haze or types of facemasks (36, 40). The compromised pulmonary function in this healthy group may be related to increased dead space, reduced ventilation, and increased inspiratory time (36).

It has been reported that wearing facemasks provides effective protection against aerosol-related challenges in the respiratory system, particularly depending on the grade of filtering of the mask. This is consistent with our current study that shows that wearing facemasks is useful in reducing the severity of COPD exacerbation (8). Although the study by Zhou et al. (8) demonstrates that wearing facemasks provides some protection for the general population, the study may also be used to explain that wearing facemasks is also a useful approach in dealing with air pollution and reducing potential exacerbation of respiratory symptoms in patients with COPD. However, future studies involving larger cohorts will be needed to clarify the optimal grade of facemasks and the period and frequency of mask-wearing for patients with COPD.

There was no significant difference in minute ventilation nor inspiratory time between COPD patients with and without facemasks during an incremental exertion test (36). In addition, no significant difference in blood lactate nor blood pressure was detected during exercise in the healthy cohort with and without facemasks (31), suggesting that facemasks have minimal impact on respiratory function. However, our analysis demonstrated that there were significantly higher lactate levels, but not blood pressure, in COPD patients without facemasks compared to those with facemasks during exercise to exhaustion. The decrease in arterial blood gas is of great significance to COPD patients with the evolution of illness (41). Therefore, there may be almost no impact of wearing facemasks on the respiratory function of patients with COPD under normal living conditions. Furthermore, wearing facemasks does not impact the exercise performance of healthy individuals (42, 43), as illustrated by our finding that there was no significant difference between the 6 WMT and maximum exercise tests in COPD patients with and without facemasks. However, we note the limitations of these tests in truly reflecting real-world conditions for patients with COPD and further studies will need to be carried out in the future.

pO_2_ and pCO_2_ are commonly used to determine the ventilation efficiency of the lungs (44). After extensive literature research, we note that there are almost no studies focusing on pO_2_ and pCO_2_ despite the importance of the alternation of pO_2_ or pCO_2_ to stimulate respiration spontaneously. It is desirable to explore the correlation between facemask wearing and the levels of pO_2_ or pCO_2_ in healthy and/or COPD cohorts under various physical conditions.

Apart from the study by Zhou et al. (8) which has demonstrated the benefits of wearing facemasks in minimizing inhaling polluted air, the World Health Organization (18) and National Health Commission of China (45) also strongly advised the use of facemasks during the COVID-19 pandemic to minimize transmission of the SARS-CoV-2 virus. The current systematic review and meta-analysis first summarize the changes in various physiological indexes and exercise functions in COPD patients with facemasks, providing a reference for daily activities and related clinical work during the COVID-19 pandemic, particularly for patients with COPD.

There are some limitations of our current study: First, the size of the literature is relatively small, regarding patients with COPD and the use of facemasks. Second, most of the original research quality is still relatively low, which cannot exclude publication bias without sufficient recorded data. Third, only the wearing of facemasks was taken into consideration regardless of the type of the facemasks and different interventions. We assume that the subjects were compliant in wearing or not wearing facemasks as required by the respective studies we searched for. We realize that the mask is a preventive factor. The mask efficacy depends upon the mask type, adherence by participants, and duration of wearing, which were unable to be ascertained by the current studies. In our future study, we have the following scopes to address: First, we will apply more stringent checks and controls to ensure subjects strictly adhere to wearing facial masks, offering more objective data. Second, we will extend our study in depth with a large number of literature studies and/or different regions/countries to boost the accuracy.

## Conclusion

We conclude that wearing facemasks partially impacts the respiratory functions of patients with COPD, regardless of the specific type of mask. More thorough investigations will be performed for both COPD and healthy cohorts, especially under real-world conditions including the COVID-19 pandemic, to further understand the physical and physiological effects of wearing facemasks.

## Data availability statement

The raw data supporting the conclusions of this article will be made available by the authors, without undue reservation.

## Author contributions

XC and CZ act as guarantors for the study, wrote the initial manuscript, and independently screened the literature. XW, ST, XX, YL, and JF conceived this study. XC, SI, CL, and FY developed search strategies. SI, CL, and FY extracted data and assessed the risk of bias in included studies. ST and XW proposed methodological suggestions. SB and JF revised the manuscript. All authors have read and approved the publication of the final manuscript.

## Funding

This study was supported by funding from the National Key R&D Program Precision Medicine Research (2017YFC0907202), Gansu Provincial Administration of Traditional Chinese Medicine (GZK-2019-33), Natural Science Foundation of Gansu Province (22JR5RA589) and the 2020 Science and Technology Project of Chengguan District, Lanzhou (2020-2-11-16).

## Conflict of interest

The authors declare that the research was conducted in the absence of any commercial or financial relationships that could be construed as a potential conflict of interest.

## Publisher's note

All claims expressed in this article are solely those of the authors and do not necessarily represent those of their affiliated organizations, or those of the publisher, the editors and the reviewers. Any product that may be evaluated in this article, or claim that may be made by its manufacturer, is not guaranteed or endorsed by the publisher.
